# Using deliberative techniques to engage the community in policy development

**DOI:** 10.1186/1743-8462-5-16

**Published:** 2008-07-16

**Authors:** Judy Gregory, Janette Hartz-Karp, Rebecca Watson

**Affiliations:** 1 Information Design Centre, Brisbane, Australia; 2 Curtin University Sustainability Policy (CUSP) Institute, Curtin University, Fremantle, Australia; 3 Australian Institute of Health Policy Studies (AIHPS), Department of Epidemiology and Preventive Medicine, Monash University, Melbourne, Australia

## Abstract

**Background:**

This paper examines work in deliberative approaches to community engagement used in Western Australia by the Department of Planning and Infrastructure and other planning and infrastructure agencies between 2001 and 2005, and considers whether the techniques could be applied to the development of health policy in Australia.

**Results:**

Deliberative processes were used in WA to address specific planning and infrastructure problems. Using deliberative techniques, community participants contributed to joint decision making and policy development. Outcomes from deliberative processes were seriously considered by the Minister and used to influence policy decisions. In many cases, the recommendations generated through deliberative processes were fully adopted by the Minister.

**Conclusion:**

The experiences in WA demonstrate that deliberative engagement processes can be successfully implemented by government and can be used to guide policy. The techniques can be adapted to suit the context and issues experienced by a portfolio, and the skills required to conduct deliberative processes can be fostered amongst the portfolio's staff. Health policy makers may be able to learn from the experiences in WA, and adopt approaches to community engagement that allow for informed deliberation and debate in the community about the future of Australia's health system.

## Background

This paper examines the application of deliberative techniques as a method for engaging the community in policy development. The paper reviews the deliberative approaches to community engagement used in Western Australia (WA) by the Department of Planning and Infrastructure (DPI) and other planning and infrastructure agencies between 2001 and 2005. The examples from WA are unusual because they show deliberative engagement techniques being integrated into the government policy development process [see Additional file 1]. This paper provides an overview of the techniques used in WA, explores key issues in using community engagement as part of policy development, and considers whether the techniques could be applied to the development of health policy in Australia. This paper has emerged from research undertaken as part of a larger project examining consumer engagement in Australian health policy conducted by the Australian Institute of Health Policy Studies (AIHPS).

### Defining community engagement

Community engagement is the process of involving the community in the planning and development of policies and services. In the health policy context, it is about involving community members in developing and implementing the policies that will affect them as health consumers. This includes decisions about the delivery of health services and the allocation of health budgets, and broader systemic questions about the type of health system that Australians want to have. Deliberative approaches to community engagement centre on involving the community in discussion and deliberation about issues, ideally leading to concrete proposals that can be adopted by policy makers.

### Overview of the experiences with deliberative processes in WA

The Australian Labor Party came to power in WA in 2001 with a platform to improve community input. The new Minister for Planning and Infrastructure, Alannah MacTiernan, was determined to champion community engagement as a way of encouraging joint decision making and democratic renewal. She employed Hartz-Karp (who is co-author of this paper) as a community engagement consultant, giving Hartz-Karp a brief to pioneer innovative ways to engage the community and industry in joint decision making with government. During her time as consultant to the Minister, Hartz-Karp facilitated almost 40 deliberative processes in WA.

The deliberative processes conducted in WA were all initiated by the Minister in response to a problem or opportunity within the portfolio. In each case, Hartz-Karp worked with Departmental staff to build a team for the project. The team adapted and combined a range of previously-documented engagement techniques to suit their local needs (drawing on work that had been undertaken in countries such as the USA, Canada, and the UK). For a summary of the major activities conducted in WA between 2001 and 2005, see the 'Participatory Democracy' section of the Minister's website .

Carson and Hart suggest that the WA experience with deliberative techniques may be unique:

'There is no equivalent in any other state of Australia, and possibly in the world, where a single politician has embraced [deliberative democratic processes] with such enthusiasm during her term of office. ... This situation confirms the catalytic nature of combining a skilled process champion with an enabling leader.' [1]

The examples from DPI and the other portfolio agencies are unusual because they were developed and implemented within government departments. In most overseas examples, deliberative techniques have been developed either by non-government organisations or academics who may work with government on a consultancy basis. For example, the citizens' jury, which is one of the most popular deliberative techniques, was developed in the USA by The Jefferson Center, a non-profit and non-partisan organisation. In WA, departmental staff were involved right throughout the process and in the implementation, and part of the purpose was to develop the department's ability to undertake this form of engagement.

## Literature review

The rhetoric of community engagement has been present in government language in Australia for some time. For example, at the State and Territory level there are explicit commitments to engaging with the community about health issues and health services, as evidenced through Departmental strategic plans [2]. However, recent research about community engagement in the Australian health policy sector suggests that engagement tends to be inconsistently practiced, with much engagement conducted at minimal levels with short consultation processes built around community submissions [3,4]. While consultation processes provide some opportunity for the community to contribute to the policy process, the community's input is limited, with no opportunity for two-way discussion, learning, and dialogue. Community engagement needs to go beyond traditional consultation if meaningful community input is to be achieved [5-7]. There is increasing evidence that the community can contribute in a meaningful way to policy decisions, but that this requires an interactive and deliberative approach [8,9].

### Levels of engagement

Community engagement can operate at a variety of levels, ranging from simple information gathering exercises that involve listening to the community's perspective, through to more complex processes that are built around two-way conversations, deliberation, and collaborative decision making. The continuum of engagement developed by Health Canada provides a useful tool for defining engagement levels; their five-level continuum describes a spectrum from low level to high level public involvement, and provides examples of when each level might be useful [10]. The five levels defined by Health Canada are (1) Inform/Educate, (2) Gather Information, (3) Discuss, (4) Engage, (5) Partner. Deliberative approaches to community engagement typically provide a high level of public involvement and would be positioned at Level 4 (Engage) or Level 5 (Partner) of the Health Canada continuum. In contrast, more traditional approaches to engagement – such as invitations for community submissions, surveys to gather information, public meetings, or inviting individual consumers to work on committees as consumer representatives – are clustered around the middle levels of the Health Canada continuum.

### Deliberative, inclusive, influential approaches to community engagement

Deliberative approaches to engagement are characterised by a process of reasoning, where participants are given an opportunity to reflect, discuss, question, and think [11]. Some key deliberative techniques are summarised in the appendix.

The definition used by the Deliberative Democracy Consortium is useful:

'Deliberation is an approach to decision-making in which citizens consider relevant facts from multiple points of view, converse with one another to think critically about options before them and enlarge their perspectives, opinions, and understandings [12].

Deliberation involves ordinary citizens being willing to tackle difficult, often value-laden problems. To achieve this, they need access to information from all perspectives, and the time required to question, reflect, and have dialogue, preferably with those who think differently to them. That way, they learn to understand and work through the trade-offs that are often integral to policy development.

One value of deliberative techniques is that participants are exposed to a range of perspectives [13]. Research shows that people involved in deliberation often change their attitudes as they listen and have time to reflect [14]. Participants are typically given information in advance, and may return to the discussion on numerous occasions. This means that participants can come to grips with complex issues and various arguments about issues in a way that is not typically possible through traditional consultation.

The possibility of engaging in meaningful deliberation is significantly enhanced if participation is diverse, inclusive, and descriptively representative. Engaging with a cross-section of the community, including people who are unaligned to specific interest groups, increases the likelihood of achieving a deliberative space, particularly when compared to engaging with 'ghettos of the like-minded', the 'squeaky wheels', and the 'usual suspects' (that is, the articulate, the vocal, and those with vested interests) [15].

While the like-minded tend to reinforce their shared views [16], diverse, inclusive participation has been shown to be an integral element of measured deliberation.

The other element integral to judicious deliberation is that of participant influence. Participants who understand that their deliberations will influence policy development or decision making are more likely to participate in a meaningful way [17,18]. Indeed, power sharing is more important than any other variable in terms of participant satisfaction with the engagement process [19].

In Australia, deliberative engagement is the exception, not the rule. Carson and Hartz-Karp note that, in Australia, community engagement has been institutionalised through legislation, regulation, policy, and accepted practice [17]. But they note that less-than-effective implementation has tended to result in increased community cynicism and reduced trust between communities and government agencies. While the language of consumer engagement may have changed to suggest a more deliberative approach, the practice has rarely followed suit [20]. Research examining community engagement in the health policy field has suggested that the mandated requirement for engagement is frequently interpreted as a short consultation process through community submissions; this minimal process tends to be relied on because staff are overworked and short of time, meaning that they default to the minimum [3,4].

Abelson, Forest et al note that deliberative processes are well suited to the health field because they can meet the broader objectives of stimulating debate, improving public understanding of complex issues, and encouraging consensus about health service priorities [21]. But, to date, there has been very limited experience in health with deliberative, inclusive, influential processes. Two citizens' juries were trialled in WA in 2000 and 2001, under the auspices of the Medical Council of WA [22]. In addition, a deliberative survey, the Joondalup Family Health Study, was designed and facilitated by Hartz-Karp in 2005 to consider the willingness of Joondalup residents to participate in the proposed research. But a literature review conducted for the AIHPS project about consumer engagement did not uncover any other examples of deliberative processes being used in health policy in Australia [2].

Some countries have adopted deliberative processes as part of their health policy making. For example, The Romanow Commission on the Future of Health Care in Canada used a deliberative process called ChoiceWork to engage consumers in discussions about health reform. Participants considered alterative scenarios for reform of health services and created their own vision of the health system they would like to see in 10 years' time [23]. In the USA, a rural community in Kentucky used deliberative forums to explore community concerns about health issues and health decision making. The forums led to several changes, including the formation of a community-based interest group – Citizens' Health Care Advocates [24]. In Denmark, consensus conferences have been used to advise parliament on a wide range of issues, including controversial health issues such as fertility treatment and gene therapy [25]. In the UK, large forums have been used to engage consumers in discussions about the future of the National Health Service. The forums used small-group techniques to encourage deliberation amongst participants [26].

The Australian Health Care Reform Alliance (ACHRA) has called for a large-scale deliberative engagement process to develop principles and priorities for health reform.

McBride and Korczak argue that strong government commitment is required for a rigorous, systematic, national engagement process [7]. They suggest that the process should use a variety of methods and be carried out by an independent organisation to ensure impartiality, legitimacy, and transparency.

## Methods

A discussion of the methods and techniques of community engagement is integral to understanding the differences between the deliberative experiences in WA and the traditional consultation techniques frequently used by health and other government agencies. In order to understand the methods from different perspectives, this section is based on Hartz-Karp's analysis of the methods within the planning and infrastructure portfolios, interviews with four DPI staff who participated in the work and had varying opinions of it, participant reports (accessed by Hartz-Karp), and summary reports written about the deliberative processes (available at ).

### Issues suitable for deliberative approaches

Within the portfolio, deliberative approaches were used to address specific problems identified by the department and/or the Minister. The primary purpose was to engage representative and inclusive community participants in discussion about issues and to facilitate joint decision making. There was also an underlying desire to encourage participative democracy [9,27]. The most distinctive aspect of the deliberative processes conducted in WA is the consistent application of inclusive, deliberative, influential engagement.

Staff within DPI agreed that not every issue is suitable for a deliberative approach. The techniques are time-consuming and expensive, and also require a considerable time commitment from participants. Issues most suited to a deliberative approach include those where the outcome will have a far-ranging or long-term effect (such as Dialogue with the City – a visioning and planning exercise for Perth), and issues where there is considerable community concern or division about the alternatives (such as the Albany Administrative Centre Citizens' Jury – which addressed the divided city's concerns about the location of the new centre – or the Road Train Summit – which used four consensus forums to address community concern about the incursion of long vehicles and road trains into metropolitan areas).

### The citizens engaged

Traditional approaches to engagement in both health and planning and infrastructure tend to involve mainly those people who are likely to experience an immediate impact from the policy change or project development. Distinctively, deliberative, inclusive approaches aim to engage a representative sample of the community in whatever technique is being used. At DPI and the other portfolio agencies, each process was guided by a steering committee that included community representatives working alongside government and industry representatives and any relevant stakeholder groups. One of their key tasks was to ensure the representativeness of participants. Though most of the deliberative processes were built around one-off events such as deliberative forums, there was considerable effort to ensure these events were part of a process that extended from framing the issues through to implementation. The process also aimed to include the broader population whenever possible.

Two different approaches were used to select participants for events:

• Random selection of participants, conducted by the WA Electoral Commission.

• A recruitment formula that included 1/3 participants who responded to invitations to a random sample of residents, 1/3 participants who responded to invitations to a broad range of relevant stakeholders, and 1/3 participants who responded to broadly placed advertisements.

The recruitment formula was developed as a way of ensuring inclusiveness in the process. It combined random selection with some purposive selection to ensure that key stakeholders and the broader community voice were included. The recruitment formula was successful for some events, but in some instances it created problems – particularly for controversial issues that involved divided community opinions and single-issue lobby groups. In these instances, lobby group invitees often tried to dominate the proceedings rather than share the deliberative space. To counteract this, rather than involving lobby groups and other stakeholder groups as invited participants at events, the agencies in WA moved towards full random selection of participants and involved these groups in other ways – such as expert witnesses, steering team representatives, and observers of the proceedings.

Random sampling, done by the independent WA Electoral Commission, drastically improved the likelihood of deliberation with voices that reflected the wider population. However, achieving truly demographically and attitudinally representative participation was elusive. Difficult to reach groups were often under-represented. Even including the time-poor, working majority was difficult. In most cases, the agencies did not pay participants for their time, and it is possible that this contributed to recruitment difficulties.

### Timing

While the deliberative processes were generally built around a specific one-day event, the processes themselves extended over a longer period. The lead-up to the event was typically around three months. After the event, the work extended into further planning and implementation, often over many months. Community representatives who had participated in the deliberative events were often involved in this ongoing work. DPI staff noted that the workload developed by a deliberative event is enormous, and often invisible to the community.

### Techniques

A variety of techniques were used, including citizens' juries, consensus conferences, deliberative surveys, televotes, consensus forums, multi-criteria analysis conferences, and 21st century town meetings [see Additional file 1]. The techniques were combined and adapted to suit the local context and the specific requirements of each project.

DPI staff recognised that deliberative techniques should be chosen to suit the issue being addressed. Issue definition happens first, before the engagement techniques are chosen. For example, the Project Director of Network City pointed out that a large scale project like developing a strategic plan for Perth required a large scale engagement exercise like Dialogue with the City. In contrast, more tightly focused issues may benefit from a different approach. But while there was flexibility to choose appropriate techniques, some staff felt that the techniques chosen were imposed on them by the Minister's Office. They noted there was a tendency to rely on large-scale, one-off deliberative forums and some of the staff interviewed felt that other techniques such as focus groups and surveys might have been more appropriate.

### A small-group approach to large-group events

Many of the deliberative processes implemented by the agencies in WA involved large groups of people meeting for a limited time. The Minister noted that, by including a larger, more diverse spectrum of people in deliberations, there would be a better reflection of the whole community's needs and, concomitantly, the outcomes reached would hold greater legitimacy with the broader public [9]. For example, the Road Train Summit and Scarborough Deliberative Survey had approximately 100 participants at each forum, while the Dialogue with the City forum involved approximately 1,100 participants.

But these large-group meetings consistently used a small-group approach, with participants sitting at round tables of 10 (or less) and taking part in a facilitated discussion. Small group discussion was maximised by:

• Seating people in small groups and arranging the seating in a way that maximised diversity. At many events, participants were placed next to someone that they did not know and were unlikely to agree with. For example, at the Road Train Summit, a person who lived on the freight route was seated next to a truck driver, and at the same table as a regulator and an environmentalist.

• Providing trained facilitators for each small group.

• Designing activities to encourage discussion and maximise deliberation.

• Collecting data from each small group and feeding it back to the larger group in real time. Scribes at each table entered into networked computers the key themes emerging from their group's discussion; in a back room, a 'theme team' pulled out the key themes from each group and projected them back to the entire group. The Project Director for Network City described the way that this process acted as a catalyst for further discussion in the small groups and also gave an opportunity for the forums' facilitator to direct the discussions with further questions.

### Combining qualitative and quantitative approaches

The engagement processes used in WA involved a combination of qualitative and quantitative techniques. Deliberations that were open-ended and qualitative were often supported by quantitative data collection and analysis techniques. For example, both the deliberative survey and 21^st ^Century Dialogue rely on a combination of quantitative surveys and qualitative deliberation. In one citizens' jury conducted in WA, the jurors chose to use a combination of quantitative multi-criteria analysis and qualitative deliberation.

Data collection supports both the qualitative and quantitative analysis. While consensus forums rely on butchers paper and sticky notes to record and group data, 21^st ^century dialogue uses a networked computer methodology that enables the recording and initial analysis of the small group deliberations to be relayed to the larger group virtually in real time. Participants can then quantitatively prioritise the themes developed by the room. A preliminary report recording all of the deliberation outcomes is given to participants at the end of the event.

The storage and further analysis of data generated through engagement processes was the responsibility of the Manager of Applied Research and Modelling at DPI. Over time, a set methodology was designed to achieve this.

### Providing the information needed for informed deliberation

A key principle underlying deliberative approaches to engagement is that participants are involved in informed discussion. Participants spend time learning about the issue so that they are able to discuss, question, and draw conclusions. In many cases, this means that extensive learning is required at the beginning of the deliberative process. DPI's Community Engagement Team Leader commented on the way that many participants seemed to value the education component of the deliberative events; they appreciated learning about the complexities of planning and hearing others' points of view.

For each deliberative process, a stakeholder steering team developed background material, usually with support from the agencies. For example, before the Road Train Summit's forums, participants received background papers developed by community groups, industry groups, local government, and state governments. At the forums, participants listened to a short presentation from each group, and had an opportunity to ask questions.

DPI staff noted that the work that went into developing the background material and preparing for the deliberative event was extensive. The background information needed to present a balanced view of all the arguments, in a form that was easily understandable to lay people but comprehensive enough to provide some depth. The various stakeholders needed to be provided with an equal opportunity to state their case to participants.

### Generating goodwill amongst participants

Generating goodwill amongst participants is an essential element of implementing successful deliberative engagement processes. Participants need to feel confident that everyone is participating in good faith, their input will be valued, the process is fair, and the outcomes will influence policy and be put into action. At the engagement initiatives conducted by the agencies in WA, participants' confidence was built through ongoing agency commitment to deliberative events, the seriousness with which the event was treated, and the commitment of the Minister and senior bureaucrats who were an integral part of each deliberative event from beginning to end.

### Evaluation

Each deliberative process at DPI was evaluated through written responses from participants. The evaluations were designed to check whether participants' expectations had been met, see whether participants felt the process was worthwhile, and provide feedback for future processes.

Participants' feedback consistently showed that they enjoyed the process and would be prepared to volunteer again. For example, the Dialogue with the City evaluation showed that 99.5 per cent of participants felt that the deliberations were either OK or great, and that 97 per cent would participate in a similar event again. Participants tended to express increased trust in government at the end of the process.

Measuring the effectiveness of deliberative processes is complex. Participant evaluation of the process offers one perspective on how the engagement was carried out, but it is obviously an inadequate measure of deliberative engagement. Similarly, while before and after survey data indicates whether attitudes and preferences have changed as a result of deliberation, it does not explain why or, specifically, what caused any changes in views (although later work in the portfolio has addressed this; particularly the large scale engagement exercise conducted for Fremantle Bridge). Moreover, neither method is useful to determine the extent of inclusiveness or influence of the process, nor whether the process has achieved any lasting effect in terms of social capital or participant political efficacy.

## Results

This section examines the impact that the deliberative processes conducted in WA had on policy development.

Each of the deliberative initiatives conducted in WA resulted in policy or infrastructure development. DPI's Manager of Applied Research and Modelling argued that the department would only go ahead with a deliberative engagement process if there was some value in it with regard to policy direction. The whole point of the engagement is to air an issue openly and develop policy options that are realisable. Deliberative processes allow the department to develop policy by working with community representatives, instead of the traditional approach of developing policy options within the department and then approaching the community for comment.

The deliberative approaches used by the agencies in WA involved a representative group of citizens in discussions about difficult issues that involved differing viewpoints and values, or complex issues. The participants helped to shape policies and influence outcomes, and the options that they generated were taken seriously. While this offers a high level of engagement to the community, the agencies in WA did not offer the community a decision-making opportunity nor a partnership approach. At each engagement event, the Minister publicly committed to take the community's views seriously and, in some cases, to implement the recommendations that the community gave. But decision-making power ultimately rested with the Minister. As MacTiernan pointed out:

'Regardless of the consultation processes we use, at the end of the day, it is my responsibility to make the final decisions. But I don't have all the answers and even if I did – I couldn't be sure I would get broad support for my "answers". With community ownership of the solution and the implementation, there is far greater likelihood that change can be achieved [9].'

Carson and Hart (2007) note that the level of decision-making input offered to citizens by MacTiernan is usual:

'What is remarkable about MacTiernan is the way in which she delivers on her promise to citizens – if she promises that their decision will be final, she delivers on that promise. She has demonstrated how an elected representative can integrate citizens' common sense and their willingness to learn in a manner which strengthens rather than diminishes the current political system [1].'

DPI staff agreed that policy outcomes clearly resulted from the deliberative engagement processes conducted. Some examples of these outcomes include:

• The community plan 'Network City', developed by participants before the conclusion of the Dialogue with the City process, was accepted by Cabinet and is now the planning framework for Perth. This document binds and guides all State government agencies, local government, and industry in the development of the metropolis.

• The Scarborough Deliberative Survey resulted in new height planning guidelines for coastal nodes.

• The Albany Citizens' Jury administration site recommendation was accepted by the Minister even though the WA Planning Commission had recommended otherwise.

• Over a two year period, the outcomes of the Freight Network Review were all implemented, resulting in sweeping new road freight policies that are now being applied across Australia. Participants in the process received progress reports until each of their priority recommendations had been implemented.

• The Reid Highway Extension Citizens' Jury proposed a specific road option and a series of safety measures, all of which were fully implemented.

• The Bassendean Enquiry-by-Design Dialogue's recommendations concerning a new railway station bridge and upgrades were accepted and have now been built.

• Recommendations from the Gascoyne Musters (a series of consensus forums held over several years) led to new guidelines for pastoralist leases and land use.

• The recommendations from the Bunbury Regional Open Space 21^st ^Century Dialogue formed the basis for the new plan for the region.

• Using the recommendations from consensus forums and deliberative surveys, a number of sites were planned and are now built or being developed including Leighton, Scarborough Senior High School site, and the Cockburn Sound industrial area.

## Discussion and Conclusion

These experiences in WA demonstrate that deliberative engagement processes can be successfully implemented by government and can be used to guide policy. Moreover, the techniques can be adapted to suit the context and issues experienced by a department, and the skills required to conduct deliberative processes can be fostered amongst departmental staff. Within the planning and infrastructure portfolio in WA, deliberative engagement processes have successfully been used to resolve community issues and concerns, identify key issues for policy development, generate consumer input into policy development, and inform major decisions.

The experiences at these portfolio agencies show that deliberative engagement processes require extensive commitment at all levels of the organisation. Most important is a high level champion, in this case the Minister, who believes that deliberative processes will facilitate better decision making and are worth the time and effort that they require. Champions are also needed throughout the organisation to help ensure that deliberative processes are implemented in ways that demonstrate a real commitment to thoroughly engaging with the community.

Building a commitment to deliberative processes throughout a portfolio takes time and effort. A move towards deliberation requires a culture change, and this tends to be slow to develop. At DPI, staff movement and the loss of skills and experience within the Department seemed to be a major impediment to this ongoing process. More importantly, such a culture change is rarely aligned with Departmental priorities or funding.

DPI's experiences show that, while the deliberative processes themselves may take significant time to implement, the work that they generate is often far more extensive. The outcomes from a deliberative event may generate work for many different sections of the Department, and across other government departments. This work is often slow to develop, complex, and invisible to the people who participated in the original event. It can also be difficult not to move away from participants' original consensus outcomes. Finding ways to communicate with participants – as DPI did in most cases through newsletters, updates, and a stated commitment to developing a concrete outcome – is critical for maintaining community support for the approach.

Hartz-Karp has noted that critics of deliberative processes suggest that they minimise the influence of informed experts and prioritise the views of the uninformed community [28]. Critics also suggest that they are too slow and costly, and tend to be used for political outcomes rather than to increase social capital. The examples from WA provide some insight into these issues:

• In many deliberative processes, DPI worked to include the voices of informed experts alongside the general community through the one-thirds recruitment approach. In addition, they put extensive effort into ensuring that community participants were well informed prior to the deliberation. Including the voices of informed experts is not without difficulty, particularly when those experts have a pre-determined idea about the best outcome.

• Deliberative processes are time-consuming and costly, but within the portfolio the techniques were adapted in ways that suited the time and money available. Although case study participants felt that they needed more time for each process, they felt that the money was well spent in developing a community-focused solution to the issue.

• The deliberative processes in the portfolio fulfilled a variety of purposes, including increasing social capital, allowing for informed decision making, and heightening public visibility and awareness of issues – including, as one officer noted, 'developing an element of political theatre' (Manager, Applied Research and Modelling). There is no reason to assume that democratic ideals and political outcomes can not work together.

The examples from WA show that deliberative processes can be used as part of government policy making, and there is good reason to believe that the processes could work well in health. Deliberative processes can improve the quality of decision making and engage the broad community in the policy development process. They can be used to resolve divisive issues and generate discussion about big picture policy issues.

Other disciplines, such as environmental management and urban planning, have a strong history of community engagement – both at the project level and the policy level. But even in those disciplines, deliberative approaches to engagement are seen as an innovative alternative to more traditional consultation techniques. While there is considerable experience in community engagement at the health service level in Australia, experience with community engagement about health policy is sparse and experience using deliberative processes is negligible. Health policy makers may be able to learn from the experiences in other disciplines, and experiences overseas with community engagement about health (particularly in Canada and the UK), and adopt approaches to community engagement that allow for informed deliberation and debate within the community about the future of Australia's health system. At a time when calls for health system reform are gathering momentum, Australian policy makers may be faced with a real opportunity to develop innovative approaches to community engagement.

For the planning and infrastructure agencies in WA, an ongoing question is how deliberative processes can be institutionalised so that they become a standard element of the engagement toolbox used by the departments. In health, the questions are centred around whether and how deliberative processes can be used to guide health policy. This requires commitment from government and strategic thinking about how deliberative processes can best be applied. It is possible that a deliberative approach to engaging the community in discussion about Australia's health policy could lead to practical approaches for health reform, based on a combination of community and stakeholder input. A robust process could develop sound policy options and provide government with a strong mandate for implementation.

## Competing interests

Janette Hartz-Karp was employed as a consultant to the Minister for Planning and Infrastructure in WA during the time covered by this case study. She organised and facilitated the community engagement activities discussed in this paper. Judy Gregory was employed as a research consultant by AIHPS to conduct research into consumer engagement in Australian health policy.

## Authors' contributions

The authors contributed equally to the development of this paper. JHK conducted the work discussed in this paper. JG was employed as the principal researcher for a larger research project about consumer engagement conducted by the Australian Institute of Health Policy Studies (AIHPS), and wrote a series of case studies from which this paper is drawn. RW provided research assistance and administrative support for the AIHPS consumer engagement project.

## Supplementary Material

Additional file 1 Key deliberative techniques used in Western Australia. This is a chart describing some key deliberative techniques used in Western Australia. Click here for file
