# Supplementary material for: Using deliberative techniques to engage the community in policy development
Source: Aust New Zealand Health Policy. 2008 Jul 16;5:16. doi: 10.1186/1743-8462-5-16 (PMC2500036; doi:10.1186/1743-8462-5-16)
Supplement: Additional file 1 — Key deliberative techniques used in Western Australia. This is a chart describing some key deliberative techniques used in Western Australia. [file 1743-8462-5-16-S1.doc]

Appendix: Some of the key deliberative techniques used in WA

| **Technique** | **When useful** | **Participants** | **Description** | **WA examples** |
| --- | --- | --- | --- | --- |
| Citizens’ Jury | When an issue involves differences in values, is complex, or involves a split community, and policy-makers want to know what a thoroughly informed random sample would think. | A small random sample of the population – usually 16-22 people. | Jurors deliberate on the issue, freely ask questions of expert witnesses, determine their values, and find options and solutions that represent a common ground. The process is guided by a facilitator. | Reid Highway Exit  Albany Administration Centre |
| Deliberative Survey/Poll | When there is concern about an issue within the community, and policy-makers want to establish whether access to comprehensive information and deliberation will change the community’s views. | A random sample of the population of approximately 200 people. | A broad community survey is carried out. Random sample participants complete a survey at the start of a one-day (or longer) deliberative forum where they hear all sides of the issue. Through small team work and plenary sessions, participants learn through inquiry and discussion. At the end of the forum, they complete the survey again. Analysis of the surveys details values and preferences, and whether these have shifted following deliberation. | Scarborough Beach Precinct Development  Fremantle Harbour Limits to Growth  Joondalup Family Health Study  Future of the Fremantle Traffic Bridge  Victoria Quay Commercial Precinct |

| **Technique** | **When useful** | **Participants** | **Description** | **WA examples** |
| --- | --- | --- | --- | --- |
| Consensus Forum | When stakeholder groups strongly disagree on an issue. | A medium-to-large group of people (60 – 150) who are representative and inclusive:   - 1/3 are respondents to an invitation to a population random sample - 1/3 are respondents to public advertisements - 1/3 are stakeholder invitees. | Participants learn about the key arguments from ‘experts’ , and then work in small groups to understand each other’s views, deliberate about key issues, and seek common ground. Themes from each small group are reflected back to the larger group.  Consensus Forums provide a way for lay people to deliberate about technically complex issues together with those who are ‘expert’ in the area. | Road Train Summit  Freight Network Review  Scarborough Senior High School Redevelopment  Gascoyne Muster  Leighton Development  South West Plantation Timber Transport Forum  State Rail Heritage Strategy  Taxi Review  South West Health Community Engagement |
| 21^st^ Century Dialogue/Town Meeting | When new thinking and planning is needed and a ground-swell of support is important. | A medium-to-large group of people (50 – 1,000s) drawn from the community – either a fully random sample or a mix of participants that includes stakeholder groups, interested community members, and random sample representation.  In some cases, additional participants may be sought to represent specific minority groups. | 21^st^ Century Town Meetings involve a large-scale meeting based on small group, facilitated discussion, with networked technology to enable the room’s key themes and priorities to be broadcast to the entire room in ‘real time’. | Dialogue with the City  Dialogue with the Pilbara: Newman Tomorrow  Cockburn Vision Dialogue  Dianelle Local Area Planning  Dialogue with Greater Bunbury on Regional Open Space |

This chart is based on: Hartz-Karp, J. (2007) 21^st^ Century Dialogue – Initiatives. Available: [www.21stcenturydialogue.com](http://www.21stcenturydialogue.com/). Additional information from the URP Toolbox, maintained by the Urban Research Program, Griffith University. Available: www3.secure.griffith.edu.au/03/toolbox/index.php (Accessed December 2007).
